# Biomedical Implications of Autophagy in Macromolecule Storage Disorders

**DOI:** 10.3389/fcell.2019.00179

**Published:** 2019-09-06

**Authors:** Adina Maria Palhegyi, Elena Seranova, Simona Dimova, Sheabul Hoque, Sovan Sarkar

**Affiliations:** College of Medical and Dental Sciences, Institute of Cancer and Genomic Sciences, Institute of Biomedical Research, University of Birmingham, Birmingham, United Kingdom

**Keywords:** autophagy, autophagy inducers, selective autophagy, macromolecule storage disorders, neurodegenerative disorders, proteinopathies, lipid storage disorders, glycogen storage disorders

## Abstract

An imbalance between the production and clearance of macromolecules such as proteins, lipids and carbohydrates can lead to a category of diseases broadly known as macromolecule storage disorders. These include, but not limited to, neurodegenerative diseases such as Alzheimer’s, Parkinson’s and Huntington’s disease associated with accumulation of aggregation-prone proteins, Lafora and Pompe disease associated with glycogen accumulation, whilst lipid accumulation is characteristic to Niemann-Pick disease and Gaucher disease. One of the underlying factors contributing to the build-up of macromolecules in these storage disorders is the intracellular degradation pathway called autophagy. This process is the primary clearance route for unwanted macromolecules, either via bulk non-selective degradation, or selectively via aggrephagy, glycophagy and lipophagy. Since autophagy plays a vital role in maintaining cellular homeostasis, cell viability and human health, malfunction of this process could be detrimental. Indeed, defective autophagy has been reported in a number of macromolecule storage disorders where autophagy is impaired at distinct stages, such as at the level of autophagosome formation, autophagosome maturation or improper lysosomal degradation of the autophagic cargo. Of biomedical relevance, autophagy is regulated by multiple signaling pathways that are amenable to chemical perturbations by small molecules. Induction of autophagy has been shown to improve cell viability and exert beneficial effects in experimental models of various macromolecule storage disorders where the lysosomal functionality is not overtly compromised. In this review, we will discuss the role of autophagy in certain macromolecule storage disorders and highlight the potential therapeutic benefits of autophagy enhancers in these pathological conditions.

## The Regulation of Autophagy

**FIGURE 1:**
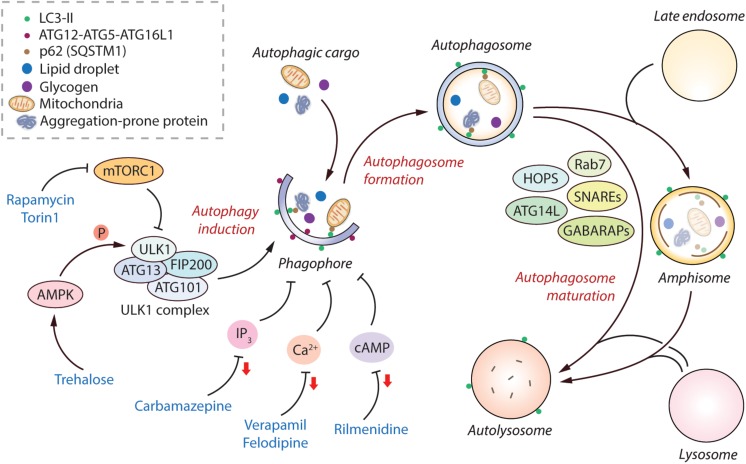
Regulation of the mammalian autophagy pathway. Autophagy initiates by the formation of phagophore that expands and engulfs autophagic cargo to form autophagosome, which then matures to form autolysosome, either by initially fusing with the late endosome to form amphisome and then with the lysosome, or by directly fusing with the lysosome. Selective autophagic cargo includes aggregation-prone proteins, damaged mitochondria, lipid droplets and glycogen, as well as the autophagy receptor protein p62; all of which are degraded in the autolysosome. Several ATG proteins including the ATG12-ATG5-ATG16L1 complex and LC3-II mediate the initiation of autophagy. LC3-II remains on the autophagosome throughout its lifespan and is thus used as a marker for autophagy. The classical regulator of autophagy is mTORC1, which negatively regulates autophagy by inhibiting the ULK1 complex. However, AMPK can positively regulate autophagy by directly phosphorylating ULK1. The mTORC1-independent regulators of autophagy include elevated intracellular levels of IP_3_, Ca^2+^, and cAMP; all of which are autophagy inhibitory signals. Autophagosome maturation in the late stage of autophagy is governed by various factors including SNAREs, HOPS complex, Rab7, GABARAPs, and ATG14L, amongst others. Autophagy can be induced pharmacologically by mTOR inhibitors (rapamycin, torin1), as well as by mTOR-independent inducers such as via AMPK activators (trehalose), and via agents lowering IP_3_ (carbamazepine), Ca^2+^ (verapamil, felodipine), and cAMP (rilmenidine) levels (reduction in second messenger molecules indicated by red arrows). Abbreviations - AMPK, 5′ adenosine monophosphate-activated protein kinase; ATG, Autophagy related; Ca^2+^, Calcium; cAMP, 3′,5′-cyclic adenosine monophosphate; FIP200, FAK family kinase-interacting protein of 200 kDa; GABARAP, γ-aminobutyric acid receptor-associated protein; HOPS, Homotypic fusion and protein sorting complex; IP_3_, Inositol 1,4,5-trisphosphate; mTORC1, Mechanistic target of rapamycin complex 1; SNARE, Soluble N-ethylmaleimide-sensitive factor activating protein receptor.

Macroautophagy (herein referred to as autophagy) is an essential process for cellular homeostasis and survival of the organism. It selectively removes undesirable macromolecules or damaged organelles which might become toxic if accumulated in the cells, and non-selectively recycles cytosolic materials during starvation to generate energy (Mizushima, 2018). This process starts with the formation of isolated membrane structures in the cytoplasm called phagophores. The origin of these membrane structures has been suggested to be from the endoplasmic reticulum (ER), as well as from other sources like mitochondria, Golgi apparatus and plasma membrane (Tooze and Yoshimori, 2010; Yu et al., 2018). The phagophores elongate to form double membrane-bound vesicles called autophagosomes, during which the autophagic cargo is engulfed. The autophagosomes then mature to form the degradative autolysosomes either via a multi-step process by initially fusing with the late endosomes to form amphisomes and then with the lysosomes, or by directly fusing with the lysosomes (Nakamura and Yoshimori, 2017). The autophagic cargo is eventually degraded in the autolysosomes by the acidic lysosomal enzymes and the breakdown products are then recycled (Figure 1). These dynamic vesicle fusion events coupled with cargo clearance are collectively defined as autophagic flux.

The classical mechanism regulating autophagy is via the mechanistic target of rapamycin complex 1 (mTORC1) pathway, which negatively regulates this process under nutrient-rich conditions (Kim and Guan, 2015). The levels of growth factors, amino acids and energy status in the cell can also influence autophagy via mTORC1 (Meijer et al., 2015). Physiologically, autophagy is stimulated by starvation via inhibition of mTORC1. Downstream of mTORC1, autophagy initiation is mediated via the ULK1 complex comprising of ULK1, FIP200, ATG13, and ATG101 (Zachari and Ganley, 2017). A positive regulator of autophagy is AMPK, which acts by either inhibiting mTORC1 or by directly inducing autophagy initiation via phosphorylation of ULK1 (Russell et al., 2014; Figure 1). Another downstream target of mTOR that indirectly prevents autophagy is the phosphorylation and cytoplasm sequestration of TFEB, which is a transcription factor mediating the expression of genes related to lysosomal function (Settembre et al., 2013). Apart from mTORC1-dependent regulation, autophagy can also be governed by mTORC1-independent pathways such as increase in the levels of intracellular inositol, IP_3_, Ca^2+^, cAMP and nitric oxide that negatively regulate this process (Sarkar, 2013; Figure 1). Several autophagy-related (ATG) proteins in the autophagic machinery mediate the autophagy initiation steps, such as the ATG12-ATG5-ATG16L1 complex and phosphatidylethanolamine-conjugated LC3-II; the latter remains associated with the autophagosomes throughout their lifespan and acts as a marker of autophagy (Mizushima et al., 2011; Ktistakis and Tooze, 2016). After autophagosome biogenesis, their lysosomal delivery and fusion are mediated by various factors including SNAREs (syntaxin 17 and SNAP29 on autophagosomes, and lysosomal VAMP8), HOPS (homotypic fusion and protein sorting) complex, Rab7, GABARAPs, BRUCE, and Beclin1-interacting partners such as ATG14L and UVRAG (Wang et al., 2016; Reggiori and Ungermann, 2017; Figure 1).

Since autophagy is implicated in myriad human diseases, small molecule autophagy modulators have potential biomedical relevance (Rubinsztein et al., 2012; Sarkar, 2013; Panda et al., 2019). Autophagy can be modulated pharmacologically either via inhibiting mTORC1 or independently of mTORC1. The commonly-used mTOR inhibitors for inducing autophagy are rapamycin (Blommaart et al., 1995) and its ester analog CCI-779 (Ravikumar et al., 2004), and Torin 1 (Thoreen et al., 2009). There are several ways of inducing mTOR-independent autophagy, such as with carbamazepine (inositol lowering agent) (Sarkar et al., 2005), trehalose (AMPK activator) (Sarkar et al., 2007a; DeBosch et al., 2016), rilmenidine (cAMP reducing agent) (Williams et al., 2008), verapamil and felodipine (Ca^2+^ channel blockers) (Williams et al., 2008; Siddiqi et al., 2019), amongst others (Figure 1). Additional means include the autophagy-inducing peptide, Tat-Beclin 1 (Shoji-Kawata et al., 2013). From a clinical perspective, since mTOR regulates critical cellular processes such as protein translation and cell growth, inducing autophagy independent of mTOR is considered to be a safer approach with lesser side-effects for biomedical applications (Rubinsztein et al., 2012; Sarkar, 2013).

## The Role of Autophagy in Macromolecule Storage Disorders

**FIGURE 2:**
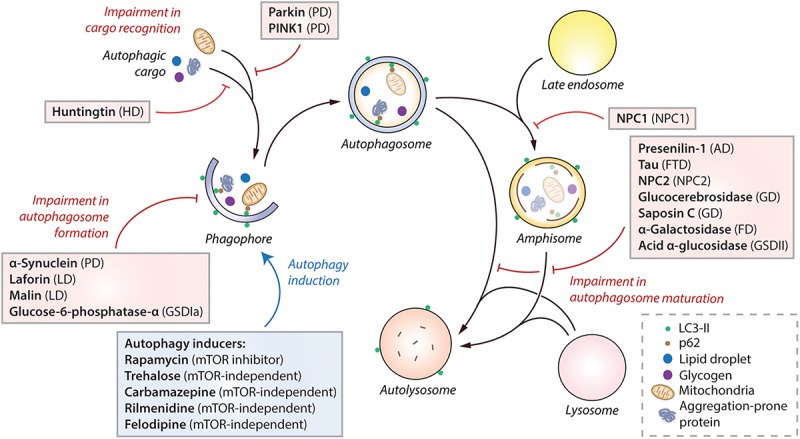
Impairment of autophagy in macromolecule storage disorders. Autophagy initiates by the formation of phagophore that expands and engulfs autophagic cargo to form autophagosome, which then matures to form autolysosome, either by initially fusing with the late endosome to form amphisome and then with the lysosome, or by directly fusing with the lysosome. Selective autophagic cargo includes aggregation-prone proteins, damaged mitochondria, lipid droplets and glycogen, as well as the autophagy receptor protein p62. LC3-II is used as a marker for autophagy as it remains on the autophagosome throughout its lifespan. Impairment of autophagy at distinct stages, such as autophagosome formation, cargo recognition and autophagosome maturation, caused due to the mutant proteins associated with various macromolecule storage disorders are indicated (red lines). Pharmacological induction of autophagy (blue arrow) by autophagy inducers (in blue box) exerts therapeutic benefits in transgenic models of many of these diseases. Abbreviations – AD, Alzheimer’s disease; FD, Fabry disease; FTD, Frontotemporal dementia; GD, Gaucher disease; GSDIa, Glycogen storage disease type Ia; GSDII, Glycogen storage disease type II; HD, Huntington’s disease; LD, Lafora disease; mTOR, Mechanistic target of rapamycin; NPC, Niemann-Pick type C; PD, Parkinson’s disease; PINK1, PTEN-induced kinase 1.

An imbalance between the production and clearance of macromolecules, causing their intracellular accumulation, can lead to a category of diseases broadly known as macromolecule storage disorders. Autophagy is a major clearance route for a wide spectrum of macromolecules such as proteins, lipids and carbohydrates, either via non-selective bulk degradation or selectively via aggrephagy (for aggregated proteins), lipophagy (for lipids), and glycophagy (for glycogen) (Stolz et al., 2014; Gatica et al., 2018). Decline in the functionality of autophagy contributes to the build-up of macromolecular materials that could be deleterious to the cells. Indeed, impairment in autophagic flux has been reported in a number of macromolecule storage disorders (Table 1). In these conditions, the defect in autophagy could occur at distinct stages: inhibition in the early stages related to autophagosome formation, or impairment in the late stages involving autophagosome maturation, lysosomal function and autophagic cargo clearance (Figure 2). Since autophagy is a vital homeostatic mechanism, compromised autophagy reduces cell viability and contributes to the disease pathology (Green and Levine, 2014; Jiang and Mizushima, 2014). Of biomedical relevance, pharmacological induction of autophagy has been shown to improve cell viability and exert beneficial effects in transgenic animal models of some macromolecular storage disorders where the lysosomal functionality is not overtly compromised (Table 1; Rubinsztein et al., 2012; Sarkar, 2013; Levine et al., 2015; Seranova et al., 2017; Panda et al., 2019). We will discuss below the role of autophagy in few macromolecular storage disorders and highlight the potential therapeutic benefits of autophagy enhancers in these conditions.

**TABLE 1:** Defective autophagy in macromolecule storage disorders and therapeutic benefits with autophagy inducers.

**Mutant proteins**	**Autophagy defects and mechanisms**	**Autophagy inducers and their mechanisms of action**	**Therapeutic benefits *in vivo* or in iPSC models**
**Proteinopathies**			

**Alzheimer’s disease (AD) and tauopathy**			

PS1 Tau	Defective autophagy due to impaired autophagosome maturation; mechanism via impairment in lysosomal acidification due to improper lysosomal targeting of v-ATPase V_0_a_1_ subunit (Lee et al., 2010; Wolfe et al., 2013) Defective autophagy possibly due to impaired autophagosome maturation; mechanism via disruption of axonal transport (Majid et al., 2014; Butzlaff et al., 2015)	Rapamycin: Induces autophagy by mTORC1 inhibition (Blommaart et al., 1995)	AD mice (*APP/Tau/PS1 mutant*) (Caccamo et al., 2010; Majumder et al., 2011), AD mice (*APP/PS1 mutant*) (Jiang et al., 2014a), AD mice (*APP mutant*) (Spilman et al., 2010), AD mice (*APOE4 mutant*) (Lin et al., 2017), FTD mice (*TDP-43*) (Wang et al., 2012), FTD mice (*tau mutant*) (Ozcelik et al., 2013; Jiang et al., 2014b; Siman et al., 2015), FTD *Drosophila* (Berger et al., 2006)
		Carbamazepine: Induces mTOR-independent autophagy by lowering inositol and IP_3_ (Sarkar et al., 2005)	AD mice (*APP/PS1 mutant*) (Tardiff et al., 2013), AD mice (*APP/Tau/PS1 mutant*) (Zhang et al., 2017), FTD mice (*TDP-43*) (Wang et al., 2012)
		Trehalose: Induces mTOR-independent autophagy via AMPK activation (Sarkar et al., 2007a; DeBosch et al., 2016)	AD mice (*APP/PS1 mutant*) (Du et al., 2013), AD mice (*APP mutant*) (Portbury et al., 2017), FTD mice (*tau mutant*) (Rodriguez-Navarro et al., 2010; Schaeffer et al., 2012)
		Latrepirdine: Induces autophagy by inhibition of mTORC1 signaling (Steele et al., 2013)	AD mice (*APP mutant*) (Steele et al., 2013)
		Gypenoside XVII (GP-17): Induces autophagy by TFEB nuclear translocation (Meng et al., 2016)	AD mice (*APP/PS1 mutant*) (Meng et al., 2016)

**Parkinson’s disease (PD)**			

α-syn	α-syn overexpression causes defective autophagy due to impaired autophagosome formation; mechanism via ATG9 mislocalization (Winslow et al., 2010) and cytoplasmic retention of TFEB (Decressac et al., 2013); whereas A53T and A30P α-syn mutants impair CMA (Cuervo et al., 2004; Martinez-Vicente et al., 2008)	Rapamycin: Induces autophagy by mTORC1 inhibition (Blommaart et al., 1995)	PD mice (*A53T*α*-syn*) (Bai et al., 2015), PD mice (MPTP treated) (Malagelada et al., 2010; Liu et al., 2013), PD mice (6-OHDA treated) (Masini et al., 2018), PD mice (L-DOPA treated) (Santini et al., 2009)
Parkin, PINK1	Defective mitophagy due to impaired targeting of damaged mitochondria to autophagosomes (Geisler et al., 2010; Narendra et al., 2010)	Felodipine: Induces mTOR-independent autophagy by lowering cytosolic Ca^2+^ (Siddiqi et al., 2019)	PD mice (*A53T*α*-syn*), A53T α-syn iPSC-derived neurons (Siddiqi et al., 2019)
		6-Bio: Induces autophagy by inhibition of mTORC1 signaling (Suresh et al., 2017)	PD mice (MPTP treated) (Suresh et al., 2017)
		Piperlongumine: Induces autophagy by Bcl2 phosphorylation and Bcl2-Beclin1 dissociation (Liu et al., 2018)	PD mice (rotenone treated) (Liu et al., 2018)

**Huntington’s disease (HD)**			

HTT	Defective autophagy due to impaired recognition of autophagic cargo (Martinez-Vicente et al., 2010) and disrupted axonal transport (Wong and Holzbaur, 2014)	Rapamycin, CCI-779: Induces autophagy by mTORC1 inhibition (Blommaart et al., 1995; Ravikumar et al., 2004)	HD mice (*HD-N171–82Q*) (Ravikumar et al., 2004), HD *Drosophila* (Ravikumar et al., 2004; Sarkar et al., 2008), HD zebrafish (Williams et al., 2008)
		Trehalose: Induces mTOR-independent autophagy via AMPK activation (Sarkar et al., 2007a; DeBosch et al., 2016)	HD mice (*HD-ex1–145Q*) (Tanaka et al., 2004)^a^
		Rilmenidine: Induces mTOR-independent autophagy by lowering cAMP (Williams et al., 2008)	HD mice (*HD-N171–82Q*) (Rose et al., 2010)
		Felodipine: Induces mTOR-independent autophagy by lowering cytosolic Ca^2+^ (Siddiqi et al., 2019)	HD mice (*HD-N171–82Q*), HD zebrafish (Siddiqi et al., 2019)
		SMER28: Induces mTOR-independent autophagy (Sarkar et al., 2007b); mechanism not known	HD *Drosophila* (Sarkar et al., 2007b)
		AUTEN-67, AUTEN-99: Induce autophagy by MTMR14 inhibition (Billes et al., 2016; Kovacs et al., 2017)	HD *Drosophila* (Billes et al., 2016; Kovacs et al., 2017)
		L-NAME: Induce mTOR-independent autophagy by NOS inhibition (Sarkar et al., 2011)	HD *Drosophila*, HD zebrafish (Sarkar et al., 2011)
		Verapamil: Induces mTOR-independent autophagy by lowering cytosolic Ca^2+^ (Williams et al., 2008)	HD *Drosophila*, HD zebrafish (Williams et al., 2008)

**Lipid storage disorders**			

**Niemann Pick type C disease (NPC)**			

NPC1	Defective autophagy due to impaired autophagosome maturation; mechanism via failure in SNARE machinery (Sarkar et al., 2013), reduction in SphK activity, and VEGF levels (Lee H. et al., 2014)	Rapamycin: Induces autophagy by mTORC1 inhibition (Blommaart et al., 1995)	NPC1 iPSC-derived neurons and hepatic cells (Maetzel et al., 2014)
NPC2	Defective autophagy possibly due to impaired autophagosome maturation (Guo et al., 2016); mechanism not known	Carbamazepine: Induces mTOR-independent autophagy by lowering inositol and IP_3_ (Sarkar et al., 2005)	NPC1 iPSC-derived neurons and hepatic cells (Maetzel et al., 2014)
		Trehalose: Induces mTOR-independent autophagy via AMPK activation (Sarkar et al., 2007a; DeBosch et al., 2016)	NPC1 iPSC-derived neurons (Maetzel et al., 2014)
		Verapamil: Induces mTOR-independent autophagy by lowering cytosolic Ca^2+^ (Williams et al., 2008)	NPC1 iPSC-derived neurons (Maetzel et al., 2014)
		BRD5631: Induces mTOR-independent autophagy (Kuo et al., 2015); mechanism not known	NPC1 iPSC-derived neurons (Kuo et al., 2015)

**Gaucher’s disease (GD)**			

GCase	Defective autophagy possibly due to impaired autophagosome maturation (Sun et al., 2010); mechanism not known but suggested via lysosomal defect (Osellame et al., 2013)	Rapamycin: Induces autophagy by mTORC1 inhibition (Blommaart et al., 1995)	GD *Drosophila* (Kinghorn et al., 2016)^b^
Sap C	Defective autophagy possibly due to impaired autophagosome maturation; mechanism not clear but suggested via inefficient cathepsin activity (Tatti et al., 2012)		

**Fabry disease (FD)**			

α-Gal A	Defective autophagy possibly due to impaired autophagosome maturation (Chevrier et al., 2010); mechanism not known	Not tested	Not tested

**Glycogen storage disorders**			

**Lafora disease (LD)**			

Laforin Malin	Defective autophagy due to impaired autophagosome formation; mechanism via mTOR activation (Aguado et al., 2010) Defective autophagy due to impaired autophagosome formation (Criado et al., 2012); mechanism not known	Metformin: Induces autophagy by AMPK activation (Buzzai et al., 2007)	LD mice (*Epm2a^–/–^*) (Berthier et al., 2016)^c^

**Von Gierke’s disease (GSDIa)**			

G6PC	Defective autophagy due to impaired autophagosome formation; mechanism via mTOR activation, AMPK inhibition (Farah et al., 2016), SIRT1 downregulation (Cho et al., 2017)	Rapamycin: Induces autophagy by mTORC1 inhibition (Blommaart et al., 1995) Bezafibrate: PPAR agonist but autophagy-inducing mechanism not clear (Waskowicz et al., 2019)	GSDIa mice (*G6pc^–/–^*) (Farah et al., 2016, 2017); GSDIa dogs (Farah et al., 2016) GSDIa mice (*G6pc^–/–^*) (Waskowicz et al., 2019)

**Pompe disease (GSDII)**			

GAA	Defective autophagy possibly due to impaired autophagosome maturation (Raben et al., 2008; Nascimbeni et al., 2012); mechanism not known	Rapamycin: Induces autophagy by mTORC1 inhibition (Blommaart et al., 1995)	GSDII mice (*Gaa^–/–^*) (Ashe et al., 2010)

**AD, Alzheimer’s disease; AMPK, 5′ adenosine monophosphate-activated protein kinase; APP, Amyloid precursor protein; AUTEN, Autophagy enhancer; Ca^2+^, Calcium; cAMP, 3′,5′-cyclic adenosine monophosphate; FD, Fabry disease; FTD, Frontotemporal dementia; α-Gal A, α-galactosidase A; GAA, Acid α-glucosidase; GCase, Glucocerebrosidase; GD, Gaucher disease; G6PC, Glucose-6-phosphatase-α; GSDIa, Glycogen storage disease type Ia; GSDII, Glycogen storage disease type II; HD, Huntington’s disease; HTT, Huntingtin; IP_3_, Inositol 1,4,5-trisphosphate; iPSC, Induced pluripotent stem cells; LD, Lafora disease; MPTP, 1-methyl-4-phenyl-1,2,3,6-tetrahydropyridine; MTMR14, Myotubularin related protein 14; mTORC1, Mechanistic target of rapamycin complex 1; NOS, Nitric oxide synthase; NPC, Niemann-Pick type C; 6-OHDA, 6-hydroxydopamine; PD, Parkinson’s disease; PINK1, PTEN-induced kinase 1; PPAR, Peroxisome proliferator-activated receptor; PS1, Presenilin-1; Sap C, Saposin C; SMER, Small molecule enhancer of rapamycin; SphK, Sphingosine kinase; α-syn, α-synuclein; TDP-43, Transactive response DNA binding protein 43 kDa; TFEB, Transcription factor EB; VEGF, Vascular endothelial growth factor. ^*a*^Protective effects of trehalose could be due to its chaperone-like ability to stabilize unfolded proteins (Tanaka et al., 2004). ^*b*^Rapamycin was cytotoxic in GD iPSC-derived neurons (Awad et al., 2015). ^*c*^Protective effects of metformin in LD may not be due to autophagy induction (Berthier et al., 2016).**

## The Role of Autophagy in Proteinopathies

Accumulation of aggregated proteins in the brain causes neurodegenerative disorders that are collectively termed proteinopathies. Autophagy is the primary clearance mechanism for several aggregation-prone proteins, such as amyloid-β, α-synuclein and huntingtin, associated with Alzheimer’s, Parkinson’s and Huntington’s disease, respectively (Nixon, 2013; Menzies et al., 2017); a process termed aggrephagy (Gatica et al., 2018). The misfolded proteins that are targeted for autophagic degradation are ubiquitinated (Grumati and Dikic, 2018). The autophagy cargo receptors, p62 (SQSTM1) (Pankiv et al., 2007), NBR1 (Kirkin et al., 2009), optineurin (Korac et al., 2013), and TOLLIP (Lu et al., 2014), recruit the ubiquitinated cargo to the autophagosomes via their respective ubiquitin-binding domain and LC3-interacting region (LIR), whereas WDFY3 (also known as ALFY) acts as a scaffold for aggrephagy (Filimonenko et al., 2010). Importantly, basal autophagy is critical for maintaining cellular homeostasis in post-mitotic neurons since abrogation of this process in normal mouse brain led to neurodegeneration (Hara et al., 2006; Komatsu et al., 2006); implicating that defective autophagy in neurodegenerative diseases is a major contributing factor. While autophagy dysfunction has been reported in almost all neurodegenerative diseases studied, few common proteinopathies where autophagy induction could be beneficial are discussed below.

### Alzheimer’s Disease and Tauopathies

The commonest cause of progressive dementia is Alzheimer’s disease (AD), characterized by abnormal metabolism of amyloid precursor protein (APP) that leads to the formation of extracellular senile plaques made of amyloid-β (Aβ) (Selkoe and Hardy, 2016). Autophagy has a role in the generation, clearance and secretion of Aβ: Aβ has been suggested to be generated inside autophagosomes containing APP and the enzyme presenilin-1 (PS-1) that cleaves APP into Aβ; Aβ is degraded via autophagy; and Aβ is possibly secreted to extracellular space via autophagy where it forms plaques (Yu et al., 2005; Nilsson et al., 2013; Tian et al., 2013). In addition, autophagy is dysregulated at multiple stages depending on the gene mutation or the disease agent in AD (Nixon, 2013). Mutations in *PS-1* gene, encoding for presenilin-1, impaired lysosomal acidification and function that blocked autophagic flux and prevented autophagosome maturation (Figure 2) because PS1 is required for targeting of v-ATPase V_0_a_1_ subunit to the lysosomes for maintaining lysosomal pH (Lee et al., 2010; Wolfe et al., 2013). Indeed, accumulation of autophagosomes was seen in AD patient brain and in dystrophic neurites of PS1/APP mice (Boland et al., 2008). On the contrary, decrease in beclin-1, which regulates autophagosome formation, was found in the affected areas of AD patient brain that could be due to activated caspase-3 cleavage (Pickford et al., 2008; Rohn et al., 2011).

The presence of intracellular neurofibrillary tangles made of hyper-phosphorylated tau, a microtubule-associated protein, is another hallmark of AD and also for tauopathies including frontotemporal dementias (FTDs) (Spillantini and Goedert, 2013). Mutant tau possibly retards autophagosome–lysosome fusion (Figure 2) by impairing the retrograde axonal transport via the dynein/dynactin complex, as seen in FTD flies and mice (Majid et al., 2014; Butzlaff et al., 2015). In addition, mutant tau colocalized with autophagic vesicles and the aggrephagy receptor protein p62 (Piras et al., 2016), and is likely degraded by autophagy (Berger et al., 2006; Wang et al., 2009).

Chemical or genetic upregulation of autophagy has been shown to be beneficial in transgenic models of AD and tauopathy. Rapamycin reduced Aβ plaques and tau tangles and rescued their pathology in APP/Tau/PS1 mutant (3xTg-AD) mice (Caccamo et al., 2010; Majumder et al., 2011), APP/PS1 mutant mice (Jiang et al., 2014a), APP mutant mice (Spilman et al., 2010), APOE4 mutant mice (Lin et al., 2017), FTD mice (Wang et al., 2012), and mutant tau mice (Ozcelik et al., 2013; Jiang et al., 2014b; Siman et al., 2015) and *Drosophila* (Berger et al., 2006). One of the studies indicated that rapamycin was effective at early stages but not after the plaques and tangles were established (Majumder et al., 2011). Likewise, the mTOR-independent autophagy enhancers, trehalose and carbamazepine, were also effective in clearing these toxic species and improving the disease pathology. Trehalose was beneficial in APP/PS1 mutant mice (Du et al., 2013), APP mutant mice (Portbury et al., 2017) and mutant tau mice (Rodriguez-Navarro et al., 2010; Schaeffer et al., 2012), while carbamazepine in APP/PS1 mice (Tardiff et al., 2013), 3xTg-AD mice (Zhang et al., 2017), and FTD mice (Wang et al., 2012). Additional autophagy-inducing compounds, such as SMER28, latrepirdine and gypenoside XVII (GP-17), were also shown to reduce Aβ levels and improve AD pathology (Tian et al., 2011; Steele et al., 2013; Meng et al., 2016). Genetically, overexpression of beclin-1 or TFEB reduced the levels and pathology of Aβ and tau in APP mutant mice (Pickford et al., 2008) or in mutant APP/PS1 and mutant tau mice (Polito et al., 2014; Xiao et al., 2014), respectively. As a likely consequence of disrupted autophagy, mitophagy is also affected in AD and is associated with mitochondrial dysfunction and bioenergetics deficits (Kerr et al., 2017); whereas stimulation of mitophagy rescued the AD pathology in APP/PS1 mutant mice (Fang et al., 2019). Overall, multiple studies have pointed that stimulating autophagy could be beneficial in AD.

### Parkinson’s Disease

Parkinson’s disease (PD) is the second most common neurodegenerative disorder characterized by motor deficits and cognitive decline (Baizabal-Carvallo and Jankovic, 2016). The commonly-mutated genes in familial PD encode for α-synuclein, PINK1, Parkin, and LRKK2, which have been shown to deregulate autophagy and mitophagy that contribute to the underlying neurodegeneration (Karabiyik et al., 2017). The α-synuclein inclusions, also called Lewy bodies that are hallmark of PD, impaired autophagosome maturation without affecting lysosomal function (Tanik et al., 2013). However, overexpression of α-synuclein (comparable to its gene multiplication in PD) in cells and transgenic mice suppressed autophagosome formation (Figure 2) by mislocalization of ATG9 (Winslow et al., 2010), and also by cytoplasmic retention of TFEB (Decressac et al., 2013). Similar trafficking defect of ATG9 resulting in autophagy inhibition was seen with mutant VPS35, which causes an autosomal dominant form of PD (Zavodszky et al., 2014). Additionally, mutations or depletion of PD-associated LRRK2, ATP13A2, and STY11 have been suggested to impair autophagy (Bento et al., 2016; Manzoni and Lewis, 2017). The A53T and A30P point mutants of α-synuclein, however, mediated their toxic effects by impairing another form of autophagy called chaperone-mediated autophagy (CMA), which involves direct protein translocation across lysosomal membrane (Cuervo et al., 2004; Martinez-Vicente et al., 2008). Apart from the defects in autophagy and CMA in PD, compelling studies demonstrate a direct role of PD-associated mutant proteins in disrupting mitophagy. PINK1 and Parkin, which are commonly mutated in autosomal recessive juvenile PD, normally functions in maintaining mitochondrial quality control (Youle and Narendra, 2011). Damaged mitochondria accumulate PINK1 on its outer membrane that recruits and activates the E3 ubiquitin ligase Parkin, which then ubiquitinates outer mitochondrial membrane proteins to initiate selective mitophagy (Pickrell and Youle, 2015). Pathogenic mutations in PINK1 and Parkin impair mitophagy (Figure 2), which is associated with mitochondrial dysfunction, oxidative stress and mitochondrial DNA mutations (Bender et al., 2006; Geisler et al., 2010; Narendra et al., 2010; Bose and Beal, 2016).

Despite autophagy defects in PD, induction of autophagy could promote autophagic clearance of α-synuclein mutants (Webb et al., 2003), and has been shown to be beneficial in toxin-induced or transgenic mouse models of PD. Genetic activation of autophagy in α-synuclein transgenic mice or rats by Beclin-1 or TFEB overexpression, respectively, decreased α-synuclein levels and ameliorated neuropathology (Spencer et al., 2009; Decressac et al., 2013). Pharmacologically, rapamycin rescued disease progression, neuronal loss and mitochondrial dysfunction in MPTP-treated PD mouse models (Malagelada et al., 2010; Liu et al., 2013) and A53T α-synuclein transgenic mice (Bai et al., 2015), improved non-motor behavioral changes in 6-OHDA treated PD mouse model (Masini et al., 2018), and prevented dyskinesia in L-DOPA-treated mouse model of Parkinsonism (Santini et al., 2009). An additive effect of rapamycin and trehalose has been demonstrated in inducing autophagy, which was neuroprotective in MPTP-treated PD mice (Pupyshev et al., 2019). Although trehalose could induce autophagy in human and other mammalian cell lines, mouse brain and iPSC-derived neurons, and has been robustly demonstrated to be effective against neurodegeneration by various studies (Hosseinpour-Moghaddam et al., 2018), a recent report indicated that trehalose and other disaccharides could block autophagy and increase α-synuclein aggregation in immortalized human cells or rat cortical neurons (Yoon et al., 2017). Nonetheless, trehalose and several other mTOR-independent autophagy inducers like verapamil, rilmenidine, valproate and calpastatin could enhance the clearance of α-synuclein mutants (Webb et al., 2003; Sarkar et al., 2007a; Williams et al., 2008). Another autophagy inducer called 6-Bio, which acts by inhibiting mTORC1 signaling, enhanced α-synuclein clearance and ameliorated toxicity in MPTP-treated PD mice (Suresh et al., 2017). Other autophagy-inducing compounds like metformin, piperlongumine and nilotinib were also protective in PD mice (Hebron et al., 2014; Liu et al., 2018; Ryu et al., 2018). Interestingly, retinoic acid derivatives such as AR7, GR1 and GR2 were shown to activate CMA for lowering α-synuclein levels and improving cell viability in a PD cell model expressing α-synuclein (Anguiano et al., 2013).

### Huntington’s Disease

Huntington’s disease (HD) is a monogenic, autosomal dominant, neurodegenerative disorder characterized by motor and cognitive deficits. HD is caused by expansion of CAG repeats in *HTT* gene, resulting in polyglutamine-expanded mutant huntingtin protein that is aggregation-prone, cytotoxic (Zoghbi and Orr, 2000), and is predominantly degraded by autophagy (Ravikumar et al., 2002). Despite accumulation of autophagosomes in HD models (Kegel et al., 2000; Petersen et al., 2001), defective autophagy in HD is attributed to failure in autophagic cargo recognition. Mutant huntingtin impairs the recognition and recruitment of autophagic cargo by autophagosomes (Martinez-Vicente et al., 2010; Figure 2), whereas wild-type huntingtin acts as a scaffold for recruiting autophagic proteins during selective autophagy such as aggrephagy and mitophagy (Ochaba et al., 2014; Rui et al., 2015). In addition, mutant huntingtin was also reported to perturb the axonal transport of autophagosomes that affected cargo degradation (Wong and Holzbaur, 2014), which was also retarded by decreased dynein function that prevented the clearance of aggregation-prone proteins and augmented toxicity (Ravikumar et al., 2005). Although mutant huntingtin is an established autophagy substrate (Sarkar and Rubinsztein, 2008), a recent study suggested that the conformation of mutant huntingtin dictates its ability to be cleared by autophagy. A toxic form of mutant huntingtin, recognized by the polyglutamine antibody 3B5H10, was found to lack Lys63 polyubiquitination that is essential to interact with p62/SQSTM1 for selective autophagy (Fu et al., 2017).

Nonetheless, pharmacological stimulation of autophagy enhances the clearance of mutant huntingtin preferentially over its wild-type counterpart (Ravikumar et al., 2002), and the beneficial effects of autophagy induction are robustly demonstrated in cell, *Drosophila*, zebrafish and mouse models of HD (Sarkar et al., 2009). Amelioration of disease phenotypes in HD mice with autophagy-inducing compounds has been shown with rapamycin (Ravikumar et al., 2004), trehalose (Tanaka et al., 2004), rilmenidine (Rose et al., 2010), calpastatin (Menzies et al., 2015), and recently, with the anti-hypertensive drug felodipine at pharmacokinetics similar to its conventional application in humans (Siddiqi et al., 2019); and also in HD fly models with SMERs (Sarkar et al., 2007b), L-NAME (Sarkar et al., 2011), AUTEN-67 (Billes et al., 2016), AUTEN-99 (Kovacs et al., 2017), verapamil and valproate (Williams et al., 2008). A combination strategy for maximal stimulation of autophagy via mTOR inhibition and mTOR-independent routes was more effective in HD *Drosophila* than either approach (Sarkar et al., 2008). Overall, modulation of autophagy presents an attractive treatment option for HD patients.

## The Role of Autophagy in Glycogen Storage Disorders

The selective clearance of carbohydrates such as glycogen via autophagy is termed glycophagy, which plays a crucial role in glucose homeostasis (Zirin et al., 2013; Zhao et al., 2018). The starch-binding domain-containing protein 1 (STBD1) has been identified as the receptor for selective glycophagy. STBD1 captures glycogen particles to autophagosomes by interacting with glycogen via its glycan-binding domain and with GABARAPL1 via its LIR motif (Jiang et al., 2011; Zhu et al., 2014). Improper glycophagy in glycogen storage disorders may contribute to the disease pathology (Zhao et al., 2018).

### Lafora Disease

Lafora disease (LD) is an autosomal recessive myoclonus epilepsy characterized by neurodegeneration and intracellular inclusions of poorly-branched glycogen deposits called Lafora bodies (Nitschke et al., 2018). The commonly-mutated genes are *EPM2A* and *EPM2B* that encode laforin (phosphatase) and malin (E3 ubiquitin ligase), respectively, which together functions in the ubiquitin-proteasome system for promoting misfolded protein clearance (Garyali et al., 2009). Knockout of these genes in mice inhibited autophagosome formation (Figure 2), suggesting impairment of autophagy in LD that was also observed in patient fibroblasts (Aguado et al., 2010; Criado et al., 2012; Duran et al., 2014). Mutant laforin suppressed autophagy by activating mTOR, whereas overexpression of wild-type laforin induced autophagy by inhibiting mTOR (Aguado et al., 2010). Activation of mTOR in laforin-deficient cells further activated serum/glucocorticoid-induced kinase 1 (SGK1), and suppression of SGK1 activity could lower glycogen accumulation, inhibited mTOR and rescued the autophagy defect (Singh et al., 2013). Defective autophagic flux could lead to inefficient glycophagy and build-up of Lafora bodies that have neurotoxic effects (Duran et al., 2014); additionally, was also associated with impaired mitophagy, increased mitochondrial fragmentation and ROS production, and decreased ATP levels in laforin- and malin-deficient cells (Roma-Mateo et al., 2015; Upadhyay et al., 2017; Lahuerta et al., 2018).

Expression of the wild-type or phosphatase-inactive mutant of laforin corrected glycogen branching, restored functional autophagy and reduced LB formation in *Epm2a^–/–^* mice (Gayarre et al., 2014), suggesting that abnormal glycogen accumulation in LD is likely via malfunction of cellular proteostasis and not due to glycogen dephosphorylation by laforin. These studies implicate defective autophagy in the pathogenesis of LD. Although a study has shown that metformin (AMPK activator and autophagy inducer) reduced LBs and neurodegeneration in *Epm2a^–/–^* mice, the beneficial effects of this compound were not attributed to autophagy induction because it did not affect autophagy *in vivo* (Berthier et al., 2016). Nonetheless, since autophagy induction could have potential benefits by facilitating LB clearance and delaying neurodegeneration, further drug trials in LD mice are required to demonstrate whether this would be a therapeutic strategy.

### Pompe Disease

Pompe disease, also known as glycogen storage disease type II (GSDII), is an autosomal recessive glycogen storage disorder that affects muscle and nerve cells, and cause muscle weakness. GSDII is caused by mutations in the gene encoding for acid α-glucosidase (GAA), a lysosomal glycogen hydrolase. GAA normally degrades glycogen, but its mutations lead to glycogen accumulation in the lysosomes especially in cardiac and skeletal muscles (Raben et al., 2002). Accumulation of autophagosomes and autophagy substrates as well as vacuolization, enlarged lysosomes and improper lysosomal acidification were found in the myotubes of patients and primary myoblasts of *Gaa*-deficient mice (Fukuda et al., 2006b; Raben et al., 2008; Takikita et al., 2009; Nascimbeni et al., 2012); implicating a block in autophagy at late stage (Figure 2). This is associated with inefficient mitophagy and impaired mitochondrial function (Lim et al., 2015). Moreover, since autophagy has been shown to influence GAA maturation and glycogen clearance (Nascimbeni et al., 2012; Zirin et al., 2013; Zhao et al., 2018), dysfunctional autophagy probably contributes to the pathogenesis of GSDII. In addition, mTOR activity was suppressed in the myotubes of *Gaa*-deficient mice where mTOR remained on the lysosomes even during starvation; and these effects including autophagosome accumulation were rescued by genetic reactivation of mTOR (Lim et al., 2017).

A common treatment for GSDII is the enzyme replacement therapy (ERT) (Desnick and Schuchman, 2012), but its efficiency is variable and temporary that has been suggested to depend on the degree of autophagy dysfunction. This is because the trafficking and processing of the recombinant human GAA seems to be affected by autophagy (Fukuda et al., 2006a; Nascimbeni et al., 2015). Autophagy activation by itself, either genetically via TFEB or TFE3 overexpression or pharmacologically with rapamycin, reduced glycogen and autophagosome load by promoting autophagosome maturation and lysosomal exocytosis in the myotubes of GSDII mice and patients and in iPSC-derived skeletal muscles (Ashe et al., 2010; Medina et al., 2011; Nascimbeni et al., 2012; Spampanato et al., 2013; Martina et al., 2014; Sato et al., 2016). Therefore, combination of ERT with autophagy induction may be more effective. Indeed, combining rapamycin or CCI-779 treatment with ERT reduced muscle glycogen levels in GSDII mice more than either treatment alone (Ashe et al., 2010). These studies highlight the therapeutic importance of autophagy in GSDII.

### Von Gierke’s Disease

Von Gierke’s disease, also known as glycogen storage disorder type Ia (GSDIa), is an autosomal recessive disorder associated with hepatomegaly and kidney failure with symptoms including hypoglycemia, lactic acidosis and hyperlipidemia. GSDIa is characterized by glucose-6-phosphatase-α (G6PC) deficiency and decreased ability of the liver and kidney to break down glycogen into glucose that causes build-up of glycogen and triglycerides (Chou et al., 2010). Autophagy has been shown to be suppressed (Figure 2) in hepatic cell and mouse (*G6pc^–/–^*) models of GSDIa possibly via activation of mTOR, inhibition of AMPK and reduced nuclear localization of TFEB (Farah et al., 2016). Likewise, increased acetyl coenzyme A in GSDIa could also have the same effect (Chou et al., 2010; Marino et al., 2014). Additionally, *G6pc^–/–^* mice also exhibited defective autophagy in the liver via downregulation of sirtuin-1 (SIRT1) (Cho et al., 2017), an enzyme which stimulates autophagy directly by deacetylation of ATG proteins and indirectly by deacetylation and activation of FoxO transcription factors that transactivate autophagy genes (Ng and Tang, 2013). Indeed, impaired autophagy in *G6pc^–/–^* mouse liver was attributed to increased acetylation of Atg5 and Atg7, decreased Atg5-Atg12 conjugation and reduced autophagosome formation (Cho et al., 2017).

Genetically, replacement of G6pc via recombinant adeno-associated virus (rAAV) vector improved hepatic glucose homeostasis and autophagic flux, and also restored SIRT1-FoxO signaling in *G6pc^–/–^* mice; although rAAV-mediated hepatic SIRT1 expression only rescued the autophagy abnormalities but not the metabolic defects (Cho et al., 2017). Pharmacologically, inducing autophagy with rapamycin reduced hepatic triglyceride, glycogen content and necrosis in the liver of *G6pc^–/–^* mice, diminished liver size and markers of liver damage in GSDIa dogs that were homozygous for an inactivating mutation in *G6pc* (Farah et al., 2016), and decreased ER stress in the kidney of *G6pc^–/–^* mice (Farah et al., 2017). In adult (older) mice, however, rapamycin could not rescue the defective autophagic flux in the liver but only marginally increased autophagosome formation (Cho et al., 2017). This study suggested that the differences between these two responses to rapamycin in the liver is thought to be due to the age of the mice as the hepatic gene expression regulated by mTOR differs depending on developmental stages and age (Boylan et al., 2015; Cho et al., 2017). Furthermore, a pan-PPAR (peroxisome proliferator-activated receptor) agonist called bezafibrate, a lipid-lowering drug used for treating hyperlipidemia, was shown to reduce liver triglyceride and glycogen levels, induce mitochondrial biogenesis and improve autophagic flux in *G6pc^–/–^* mice (Waskowicz et al., 2019). Since PPARα agonists such as Wy-14 643 and GW7647 could stimulate hepatic lipophagy and attenuate liver injury in mice and patients with acute liver failure (Jiao et al., 2014; Lee J.M. et al., 2014), modulation of PPARα (a nutrient-sensing nuclear receptor) for inducing hepatic autophagy might represent a potential therapeutic target for the treatment of GSDIa.

## The Role of Autophagy in Lipid Storage Disorders

Autophagy is a major clearance route for intracellular lipids via a process termed lipophagy. This is evident from genetic ablation of autophagy in mice that increased triglyceride content and lipid droplets (Singh et al., 2009). However, the exact mechanism of selectively targeting the lipid droplets for autophagic degradation is not clear. In lipid storage disorders, malfunction of autophagy could thus contribute to the disease pathology (Ward et al., 2016).

### Gaucher Disease

Gaucher disease (GD) is an autosomal recessive lysosomal storage disorder associated with neurological damage and hepatosplenomegaly. GD is characterized by mutations in *GBA1* gene encoding for glucocerebrosidase (GCase), an enzyme that hydrolyses glucosylceramide; and in rare cases by mutations in *PSAP* gene that leads to deficiency of the GCase activator, saposin C (Stirnemann et al., 2017). Lysosomal accumulation of glucosylceramide and glucosylsphingosine in GD is thought to impair autophagy in the late stages (Figure 2). Indeed, a block in autophagic flux associated with accumulation of autophagy substrates, ubiquitinated proteins, autophagosomes, and inefficient cathepsin activity, was seen in mice, flies, patient fibroblasts and iPSC-derived neuronal models of GD with GCase or saposin C deficiency (Sun et al., 2010; Vaccaro et al., 2010; Tatti et al., 2012; Osellame et al., 2013; Farfel-Becker et al., 2014; Awad et al., 2015; de la Mata et al., 2015). Defective mitophagy, mitochondrial dysfunction and oxidative stress were also found in GD that are likely consequences of impaired autophagy (Cleeter et al., 2013; Osellame et al., 2013; de la Mata et al., 2015; Kinghorn et al., 2016; Li et al., 2019).

Interestingly, mutations in *GBA1* gene could induce neurodegeneration and correlated with increased risk of developing Parkinson’s disease (PD) (Sidransky et al., 2009; Farfel-Becker et al., 2014). This is because GCase and α-synuclein form a pathogenic loop wherein GCase deficiency (in GD) caused autophagic dysfunction and α-synuclein accumulation whereas overexpression of α-synuclein (in idiopathic PD) could disrupt GCase trafficking and activity (Mazzulli et al., 2011; Schondorf et al., 2014; Du et al., 2015; Magalhaes et al., 2016). In a *Drosophila* model of neuronopathic GD, decreased mTOR activity and upregulation of the fly ortholog of mammalian TFEB was observed, which is thought to work as a compensatory mechanism for overcoming the autophagic block (Kinghorn et al., 2016). Further inhibition of mTOR with rapamycin extended the life span and rescued the disease pathogenesis in GD flies (Kinghorn et al., 2016). Contrary to these fly data, GD patient-specific iPSC-derived neurons exhibited downregulation of TFEB and lysosomal genes, where rapamycin treatment caused cell death (Awad et al., 2015). However, recombinant GCase rescued the defective autophagy and lysosomal depletion phenotypes in these disease-affected neurons; effects that were augmented by TFEB overexpression but not on its own (Awad et al., 2015). It is plausible that induction of autophagy in the presence of lysosomal dysfunction might be detrimental in disease-affected neurons, however, various mTOR-independent autophagy inducers need to be assessed in GD neurons to make this conclusion.

### Niemann-Pick Type C Disease

Niemann-Pick type C (NPC) disease is an autosomal recessive, lysosomal storage disorder associated with severe neurodegeneration and hepatosplenomegaly. It is caused by mutations in *NPC1* gene (95% cases; NPC1 disease) or *NPC2* gene (5% cases; NPC2 disease) (Vanier, 2010). NPC1 (lysosomal cholesterol transporter) and NPC2 (lysosomal glycoprotein) proteins are suggested to facilitate cholesterol transport from the late endosomal/lysosomal compartments, and their mutations cause accumulation of unesterified cholesterol in the brain and other tissues (Karten et al., 2009; Kwon et al., 2009). In NPC1 and NPC2 disease, a block in autophagic flux occurs due to impaired autophagosome maturation (Figure 2) that results in build-up of autophagosomes and autophagic cargo (Sarkar et al., 2013; Lee H. et al., 2014; Guo et al., 2016) including defective mitophagy and mitochondrial function (Ordonez et al., 2012; Guo et al., 2016); although lysosomal function was not overtly compromised (Sarkar et al., 2013). Various mechanisms underlying autophagy defect in NPC1 disease have been described using mutant mice, patient fibroblasts and iPSC-derived neuronal models. These include failure in the SNARE machinery between autophagosomal Syntaxin-17 and late endosomal/lysosomal VAMP8 to prevent vesicle fusion (Fraldi et al., 2010; Sarkar et al., 2013), accumulation of sphingosine due to reduced sphingosine kinase activity and vascular endothelial growth factor (VEGF) levels (Lee H. et al., 2014), and depletion of lysosomal Ca^2+^ stores (Lloyd-Evans et al., 2008) that could affect autophagy via calcineurin or calpain (Williams et al., 2008; Medina et al., 2015). Interestingly, the most common NPC1 mutant (I1061T) is shown to be selectively degraded via ER autophagy (ER-phagy) and ER-associated protein degradation (ERAD) (Schultz et al., 2018).

Of therapeutic relevance, autophagy inducers such as rapamycin and carbamazepine rescued the autophagy defect and improved cell viability in NPC1 iPSC-derived neuronal and hepatic cells, whereas trehalose, verapamil and BRD5631 were effective only in neurons (Maetzel et al., 2014; Kuo et al., 2015). Although the multi-step route of autophagosome maturation was impaired in NPC1 disease, autophagy induction restored functional autophagic flux via the bypass mechanism enabling direct autophagosome–lysosome fusion; but this did not reduce the cellular cholesterol load (Sarkar et al., 2013). Strikingly, cholesterol-depleting agents like 2-hydroxypropyl-β-cyclodextrin, which has advanced in clinical trials, exhibited further impairment in autophagic flux, neurotoxic effects and adverse effects in animals (Peake and Vance, 2012; Sarkar et al., 2013; Vite et al., 2015). However, β-cyclodextrin derivatives such as methyl-β-cyclodextrin or β-cyclodextrin-threaded biocleavable polyrotaxanes could rescue the autophagy defect and enhance cholesterol clearance in NPC1 cells (Tamura and Yui, 2015; Dai et al., 2017); although the effect on autophagy may not be their primary roles. Future directions may include efficacy studies of combination treatment involving autophagy inducer and a low-dose of cholesterol-depleting agent in mouse models.

### Fabry Disease

Fabry disease (FD) is a X-linked lysosomal storage disorder characterized by mutations in the *GLA* gene on the X chromosome encoding the lysosomal enzyme, α-galactosidase A (α-Gal A). FD is associated with impaired glycosphingolipid metabolism, lysosomal accumulation of a particular fat called globotriaosylceramide (Gb3) and multi-organ dysfunction (Germain, 2010). Autophagic flux is impaired in FD possibly due to defective autophagosome maturation (Figure 2), as evident from accumulation of autophagosomes, lysosomes and autophagy substrates in patient fibroblasts, human podocyte model and α-Gal A deficient mice (Chevrier et al., 2010; Liebau et al., 2013; Nelson et al., 2014). However, build-up of autophagosomes may also occur due to increased biogenesis because FD podocytes displayed inhibition of mTOR and increased expression of autophagy initiation genes, *BECN1* and *GABARAP* (Liebau et al., 2013; Braun et al., 2019). Autophagy dysfunction has been suggested to contribute to the neuropathological manifestations of FD, and also to podocyte damage in the kidneys resulting in end-stage renal disease that is a common cause of death in FD (Liebau et al., 2013; Nelson et al., 2014). In female patient fibroblasts, the disease severity correlated with the extent of impaired autophagy (Yanagisawa et al., 2019). The autophagy dysfunction is thought to interfere with the efficiency of ERT in FD podocytes (Chevrier et al., 2010); a phenomenon also reported in Pompe disease. Therefore, ERT coupled with induction of autophagy could be evaluated to improve the disease outcome.

## Concluding Remarks

Autophagy is responsible for the maintenance of cellular homeostasis, and the functionality of this vital process is impaired in different ways in myriad macromolecule storage disorders associated with accumulation of aggregation-prone proteins, carbohydrates and lipids. Beyond the pathologies outlined in this review, autophagy has been implicated in several other macromolecule storage disorders including amyotrophic lateral sclerosis and polyglutamine diseases associated with build-up of aggregation-prone proteins (Menzies et al., 2017), and neuronal ceroid lipofuscinosis associated with build-up of lipofuscin that are lipopigment materials made up of lipids and proteins (Seranova et al., 2017). Defective autophagy reported in many such conditions has been suggested to contribute to the disease pathology, whereas in certain instances, stimulation of autophagy is beneficial which could reduce the macromolecular burden and prevent cytotoxicity. Autophagy inducing-compounds have been shown to attenuate the disease phenotype in various cell, fly and mouse models of these diseases where the lysosomal degradative capability if not overtly compromised. Modulation of autophagy by small molecules might thus represent an attractive therapeutic strategy in macromolecule storage disorders, but disease-specific treatments must be thoroughly researched because some of the conditions might require a combination of autophagy inducer and conventional therapy.

## Author Contributions

AP and SS wrote the overall manuscript. ES, SD, and SH reviewed and contributed to the manuscript. AP, SD, and SS made the table. AP, SH, and SS made the figures.

## Conflict of Interest Statement

The authors declare that the research was conducted in the absence of any commercial or financial relationships that could be construed as a potential conflict of interest.
